# Maternal social networks and child nutrition in Belagavi, India: opportunities and risks

**DOI:** 10.1080/16549716.2026.2741746

**Published:** 2026-10-14

**Authors:** Maja Petersson, Anusha Kulkarni, Mubashir Angolkar, Julia Schröders, Miguel San Sebastian, Masoud Vaezghasemi

**Affiliations:** a Department of Epidemiology and Global Health, Umeå University, Umeå, Sweden; b School of Public Health, KLE University, Belagavi, India

**Keywords:** Maternal social networks, child malnutrition, nutritional interventions, India, HDSS

## Abstract

**Background:**

Strengthening mothers’ social networks can help address child malnutrition and improve health outcomes. This is important in Belagavi district, India, where undernutrition and anaemia remain prevalent among children under five. However, social networks may also have unintended negative influences on child health, depending on their structure and the information and support they provide.

**Objective:**

This study examines the association between maternal social networks, defined by the number of close friends, on child nutritional outcomes in Belagavi, Karnataka, India.

**Methods:**

We used secondary cross-sectional data from the 2024 Health and Demographic Surveillance System catchment area of KAHER JNMC in Belagavi [KAHER HDSS]. A total of 595 children aged 0–61 months and their mothers were included. Descriptive analyses are reported as frequencies and percentages. Bivariable logistic regressions assessed associations between maternal social network [low < 3 friends; high ≥ 3 friends] and nutritional outcome. Multivariable logistic regressions were adjusted for child age, sex, birth order, maternal education, maternal age, and maternal occupation.

**Results:**

About half of mothers [54%] had fewer than three close friends. A strong maternal social network was associated with lower odds of stunting, wasting, never breastfeeding, and delayed initiation of breastfeeding. However, it was associated with increased odds of underweight, anaemia, inappropriate introduction of complementary foods, and low birthweight.

**Conclusions:**

Maternal social networks were associated with both favourable and unfavourable child nutritional outcomes. Public health interventions should consider the complexity of these associations when designing strategies to improve child nutrition and minimise its unintended consequences.

## Paper Context

- **Main findings**: Strong maternal social networks were protective for some child nutrition outcomes but increased the risks for others.
- **Added knowledge**: This study highlights the double-edged role of maternal social networks, showing that larger networks do not uniformly benefit child nutrition.
- **Global health impact for policy and action**: Interventions to strengthen maternal networks must account for both positive and negative effects to design context-sensitive, equitable child nutrition strategies.

## Background

Social Network Theory views social relationships as connections, known as ties, between individual actors, referred to as nodes [1–4]. These ties and nodes together form what are called social networks. The theory suggests that the structure of a social network influences people’s behaviour and attitudes by shaping the flow of resources, ultimately determining their access to opportunities and information, as well as the constraints on their actions [1–4]. The strength of a tie is assumed to be determined by factors such as the frequency and quality of contact and interaction, whether the interaction is more intimate or whether the connection provides resources such as social and emotional support. These actors are often family, close relatives, or close friends and are considered strong ties. In contrast, weak ties are described as connections that do not require as much time and effort and are sometimes the connections between clusters of strong ties. These actors are acquaintances, and they broaden the social network. The strength of weak ties lies in their ability to increase diversity within the social network and thereby potentially generate a larger social network and a greater flow of resources [5,6].

Social networks are widely recognized as contributors to both mental and physical well-being [7–9]. Because of the complexity of social networks, researchers have defined them differently and sometimes even used the term loosely or interchangeably with related concepts such as social support or social integration [2,4]. As a result of this, Berkman et al. presented a framework illustrating how social networks can be put into context and how several different pathways connect social networks to health, with the purpose of bringing greater conceptual clarity to the research field [1–3]. First, a social network must align with the local cultural and social context, including socioeconomic factors and the political climate. These elements shape its structure and regulate how it can be formed. The structure of a social network is characterised by many factors, including the size of the network, range, density, homogeneity or heterogeneity, and the characteristics of the network ties. This structure then creates opportunities for downstream pathways by providing opportunities for social support, social influence, social engagement, personal contact, and access to resources and goods. All of these generate health effects by influencing behaviour, generating physiological responses, or mediating psychological effects. However, all these pathways connecting social networks to health outcomes have strengths and limitations. For instance, while social support generated by a large social network may be an important contributor to good mental health, it may also facilitate the transmission of HIV and tuberculosis. Similarly, social influence may generate either a positive or negative health effect as shared norms have proven to be closely associated with smoking habits, alcohol consumption, and dietary patterns [1–4].

Adapting the theory behind this framework, changes in upstream factors cascade through social networks and generate health effects. Thus, leveraging social network interventions in public health can not only enhance individual health but also generate a wave of positive health outcomes across the entire network [7,8]. Public health interventions targeting social networks, aiming to influence and shape attitudes and behaviours within them, have been shown to be more effective in achieving lasting improvements in behavioural health outcomes [10]. This was supported by the findings of Hunter et al. in their systematic review and meta-analysis on social network interventions. Their study included 37 randomized controlled trials (RCT) assessing the effects of these interventions on health outcomes, where significant positive effects on alcohol misuse, smoking habits, sexual health, well-being, and diabetes management, as measured by changes in haemoglobin A1c (HbA1c) were reported [9]. Most of the studies included in the review were conducted in high-income countries (HICs) with only six from LMICs, highlighting the need to research social networks and their effects on health outcomes in these settings. Three of the six studies reported statistically significant findings that align with the results from HICs, showing that public health interventions within social networks successfully increased the use of contraceptives and were protective against HIV. The other studies from LMIC countries included did not have statistically significant results for decreasing drug use and associated risk behaviour or for successfully influencing product use. These results are still promising and suggest that public health interventions may have long-term effects in LMIC settings; however, their effect is dependent on the intervention target [9].

A continuing public health challenge in India is children’s health, especially child malnutrition [11]. Malnutrition is an excess or deficiency in energy or micronutrient intake, and among children under five, the most prevalent form of malnutrition is undernutrition, which includes stunting [low height-for-age], wasting [low weight-for height], and underweight [low weight-for-age] [12]. The National Family Health Survey (NFHS-5) from 2019 to 2020 reported that 36% of the children under five in India were stunted, 19% were wasted, and 32% were underweight. In comparison, with the previous report from 2015 to 2016 (NFHS-4), malnutrition in India among children under five has slightly decreased. However, childhood anaemia showed the opposite trend, increasing from 59% to 67% [13]. Risk factors for developing chronic undernutrition in children in India, such as stunting, include low household wealth, poor diet diversity, low maternal education, and maternal undernutrition (underweight or short maternal stature) [14]. These risk factors are shared across the LMIC, and maternal education and household wealth are recognized as key determinants of malnutrition in children [15]. Wasting is classified as acute undernutrition and is often caused by sudden weight loss, which may result from limited access to food or an inability to absorb nutrients due to disease. Underweight is the result of either chronic or acute undernutrition [11]. It is well known that undernourished children face an increased risk of developmental delays, infections, underweight, and anaemia [11,15].

As previously mentioned, social networks mediate the relationship between broader social contexts and individual health outcomes [1–4]. Within the family context, maternal social networks may influence child health outcomes, both positively and negatively through several interconnected mechanisms. Social ties can facilitate the exchange of health and nutrition information, promote the diffusion of beneficial childcare and feeding practices through shared social norms, provide emotional support that strengthens caregiving capacity, and improve access to material resources such as food, childcare, and financial assistance during times of need. Conversely, social networks may also reinforce harmful norms or misinformation, potentially contributing to suboptimal feeding and caregiving practices. The influence of social networks is therefore context-dependent and shaped by the availability, quality, and nature of social relationships. Previous studies have demonstrated these pathways. For example, maternal and child social support has been associated with improved household food availability and child growth across multiple LMIC settings, highlighting the importance of supportive social environments beyond household socioeconomic conditions [16]. Similarly, research from Mexico has shown that infant feeding practices are embedded within broader social networks, where advice, cultural expectations, and caregiving norms collectively shape maternal decision-making [17]. These findings suggest that maternal social networks represent an important social determinant of child nutrition that extends beyond individual maternal characteristics. Therefore, public health interventions that strengthens maternal social networks may be an important strategy for improving child health [18].

Research examining how maternal social networks affect child health in LMIC settings is limited. However, previous studies have shown that maternal social relationships are associated with child nutritional status and the uptake of maternal and child health services, suggesting that social connectedness may influence child health through multiple behavioural and resource-related pathways [19,20]. Findings from a study conducted in Andhra Pradesh, a south Indian state bordering Karnataka, showed a positive association between maternal network size and reduced stunting. The same study also found that network literacy can reduce stunting, and the association was stronger for poorer households [21]. These results suggest that knowledge shared within maternal social networks can also limit the effect of socioeconomic vulnerabilities when it comes to child undernutrition. However, evidence remains scarce regarding whether these associations extend to other dimensions of child health beyond stunting and whether they are observed in different sociocultural contexts within India. As the influence of maternal social networks on child health can be diverse and context-dependent, this study aims to examine the association between maternal social network size and multiple child health and nutritional outcomes among children aged under 5 years in Belagavi, Karnataka, India. The findings can provide valuable insights for future public health interventions in similar contexts.

## Methods

### Study context

This project was conducted in the Belagavi Taluka of Belagavi district, the second most populous district in Karnataka, India, with a population of approximately 5.4 million people. The Belagavi district has been transitioning from rural to urban and has a population with diverse socioeconomic backgrounds. The NFHS-5 reported several nutritional challenges with high rates of anaemia (73%) and malnutrition among children under five, particularly stunting (33%), wasting (24%), and underweight (37%) [22].

### Study design, data collection, and participants

The data for this project was based on a cross-sectional design using secondary data from the Health and Demographic Surveillance System catchment area of KAHER JNMC (KAHER HDSS), Belagavi, Karnataka. The KAHER HDSS is a university initiative introduced in 2023 August and data collection has continuously taken place since. KLE University is supporting four primary health centres (PHC) in Belagavi district, in collaboration with the Indian government and other private partners. Two of these PHCs are rural and two are urban and located in socioeconomically disadvantaged areas. The catchment area for this study was the population of the two rural PHCs in Kinaye and Vantamuri and included 15 rural villages. In India, a rural village is defined as a village with a population less than 5000, a population density less than 400 per square kilometre, and the occupation of more than 25% of the male population is in agriculture [23]. Some villages have because of the growth in population, exceeded 5000 but may still be considered as rural if there has been no industrial development.

Data collection for this project took place between June and September 2024 using structured household surveys and was conducted by trained field workers in the participants’ mother tongue. Responses were recorded in English on paper during the interviews and subsequently entered manually into an Excel sheet. The dataset included information on socioeconomic, demographic, household, individual, and child characteristics, from all households within the catchment area. Households were included if they had at least one child aged 0–61 months residing in the home at the time of data collection, resulting in a sample of 595 participants. There were no exclusion criteria applied during the data collection. Two children were excluded from the final analysis because the mother had deceased or there was no information about the mother. For some outcomes, missing values resulted in a slightly smaller sample size for that analysis.

### Outcome variables

The outcome variables included in this study were as follows: birthweight, breastfeeding practices, including early initiation of breastfeeding and whether the child was ever breastfed, consistency of first solids, symptoms of anaemia, and nutritional indicators such as stunting, wasting, and underweight. These variables were chosen as outcomes as they reflect different aspects of the child’s nutritional status that may be influenced by the mother. Specifically, the mother’s health status during pregnancy may reflect on the child’s birthweight, childcare practices may be revealed by breastfeeding choices and how the child was introduced to the solids. Mothers’ access to food and nutrition may be reflected in child malnutrition outcomes, including stunting, wasting, underweight, and anaemia.

To collect information on birthweight, mothers were asked about the child’s birthweight, which was recorded as <2.5 kg (i) and ≥2.5 kg (ii). To explore breastfeeding practices, mothers were asked whether their children were ever breastfed (yes/no), and if the breastfeeding was initiated within 1 hour of birth (yes/no). To explore the consistency of first solids, mothers were asked what kind of solids they first introduced to their child on the first day. Responses were recorded as liquid (i), semi-solid/soft (ii), or solid (iii). Mothers who exclusively reported semi-solid/soft were categorized as ‘correct’, while those who had given their children liquid, solid food, or varying consistency throughout the day were categorized as ‘incorrect’. To assess the prevalence of anaemia, data collectors were trained to look for the clinical symptoms of anaemia. As haemoglobin measurements were not feasible within the surveillance system, these observational signs were used as a proxy for anaemia, recognizing that they may be subject to misclassification. Data collectors examined the child for paleness of skin on the palms, nail beds, or conjunctiva, recorded as pallor (yes/no), and whether the child had koilonychia (spoon-shaped nails) (yes/no). If the child had either pallor (yes) or koilonychia (yes), the child was considered to experience symptoms of anaemia. Nutritional status was assessed using the z-score for weight-for-height and height-for-age based on the WHO growth standards. This anthropometric assessment is based on child characteristics, such as age, sex, height, and weight. The z-score indicates how far the measurement deviates from the median, with a z-score of 0 representing the median value. According to the WHO Global Database on Child Growth and Malnutrition, a z-score of −2 standard deviation (SD) was used as the cutoff to classify stunting, wasting, and underweight [24]. If the child was not home at the time when data collectors visited the home, they were able to either examine the children at the Anganwadi preschool for children aged 1–5 or personnel at the Anganwadi would examine these children and report any findings of symptoms of anaemia, height, and weight to the data collectors.

### Exposure variable

The single exposure, or main explanatory variable in this study, was the mother’s social network, conceptualised as the availability of close, supportive social ties. It was measured using the question: ‘How many close friends do you have, people that you feel at ease with and can talk to about private matters?’ Responses were structured into five categories: none (i), 1–2 (ii), 3–5 (iii), 6–9 (iv), and 10 or more (v). These were dichotomised into low (i–ii) and high (iii–v) to create categories sufficiently large for analysis. This measure captures both the size and perceived quality of the mother’s social network, a commonly used proxy for social support in epidemiological research [3,8].

### Covariates

Variables that were used to control for explanatory variables in the regression analysis included child age (months), sex (male/female), and birth order at the child level; at the household level, maternal education level, maternal age (years), and maternal occupation were included. Birth order was categorized into four groups: first-born (i), second-born (ii), third-born, or later (iii). Mother’s education was based on the highest education which they finished; no education (0), primary school (i), junior secondary school (ii), senior secondary school (iii), and higher education [iv]. No education or completion of primary school and junior secondary school were, during the analysis joint together (no or lower education).

### Analysis

The outcome-wide epidemiology design examines the effects of a single exposure and simultaneously addresses multiple outcomes. Frequencies and percentages were used to describe the general characteristics of the study participants. Simple (Crude) logistic regression was used to assess the relationship between the main explanatory variable, the social network of the mother, and each outcome variable separately first, and then a multiple (Adjusted) regression model was estimated including the potential confounding variables. The crude and adjusted model has also been presented in the form of a forest plot for better interpretability purposes. Odds ratios (OR) and 95% confidence intervals (CI) were used as measures of association and statistical inference, respectively. An advantage of outcome-wide approaches is that they allow the comparison of exposure effects across outcomes and provide a direct comparison of effect sizes [25]. The software R was used to analyse the data.

## Results

In total, there were 595 children of which 302 (51%) were boys and 293 (49%) were girls. Approximately half of the mothers, 319 (54%), had none or either one or two close friends (low) and 274 (46%) had three or more close friends (high) whom they could talk to with ease. The most prevalent child nutritional outcome was the introduction of first complementary foods as most mothers (63%) introduced foods with inappropriate texture. In contrast, good breastfeeding practices seem to be well integrated within the community and among the mothers as few children (9%) were ‘never breastfed’ or experienced ‘delayed breastfeeding initiation’ (13%). The prevalence of undernutrition was high, with 43% of the children classified as stunted, 37% as underweight, and 23% as wasted. Few study participants (15%) were born with a birth weight of less than 2.5 kg. For most outcomes, the prevalence was similar between the sexes, with the largest differences observed in stunting and anaemia. Almost half of the boys (49%) were affected by the stunting, compared to 37% of the girls. Symptoms of anaemia were present in 53% of the girls and 43% of the boys. Table 1 shows the general characteristics of the study population and the prevalence of each nutritional outcome, overall and for each sex which can also be visualized in Figure 1.

**Figure 1.**
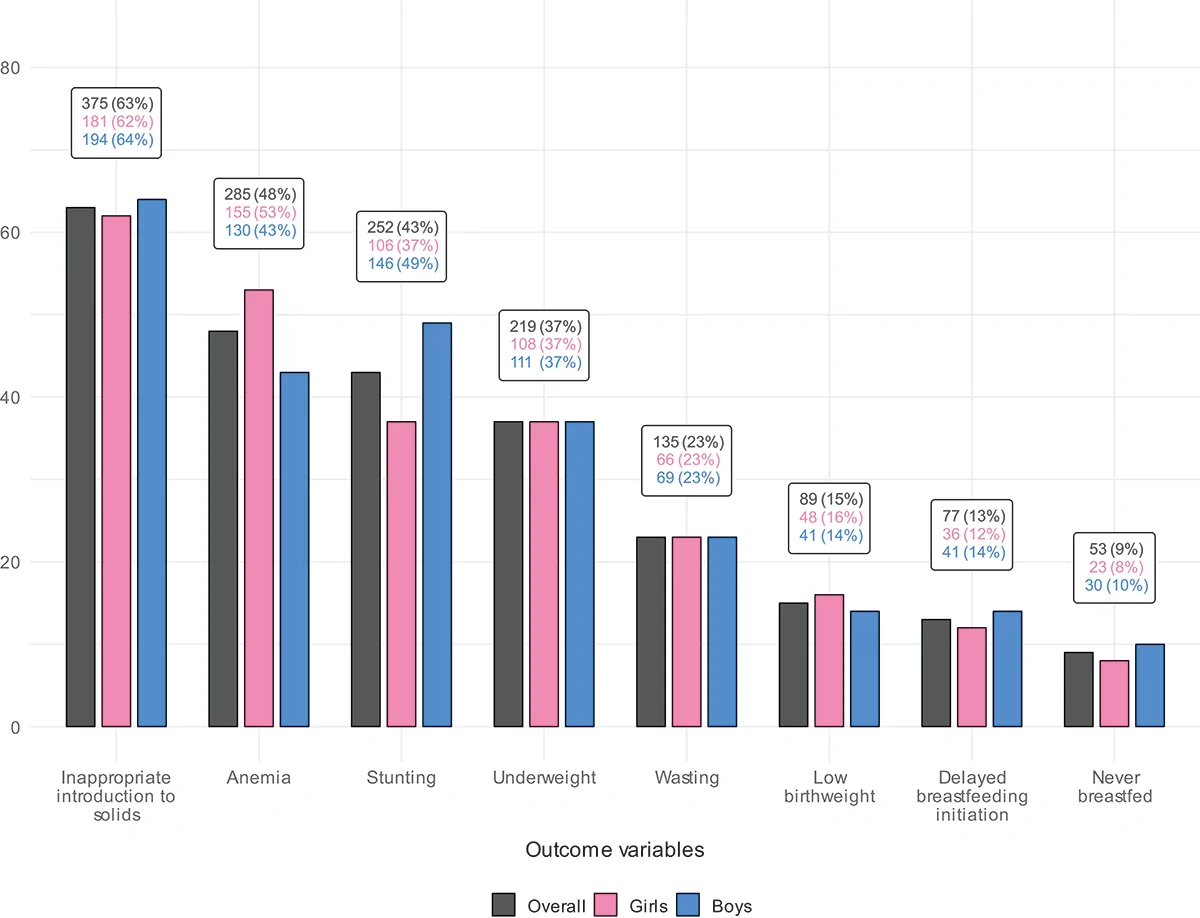
Counts and percentage for all nutritional outcomes for all children aged 0–61 months, boys and girls separately, based on HDSS, Belagavi, India 2024.

**Table 1.** General characteristics of the study population based on the HDSS, Belagavi, India, 2024.

	All n[%]	Boys n[%]	Girls n[%]	All n[%]
TOTAL	595 [100%]	302 [51%]	293 [49%]	319 [54%]
CHILD HEALTH RELATED OUTCOMES				
Symptoms of anaemia				
No	308 [52%]	171 [57%]	137 [47%]	187 [59%]
Yes	285 [48%]	130 [43%]	155 [53%]	132[41%]
Birthweight				
Normal [≥2.5 kg]	505 [85%]	260 [86%]	245 [84%]	274 [86%]
Low [<2.5 kg]	89 [15%]	41 [14%]	48[16%]	45 [14%]
Consistency of first solids				
Appropriate	220 [37%]	108 [36%]	112 [38%]	123 [39%]
Inappropriate	375 [63%]	194 [64%]	181 [62%]	196 [61%]
Underweight				
Normal	370 [63%]	189 [63%]	181 [63%]	201 [64%]
Underweight	219 [37%]	111 [37%]	108 [37%]	113 [36%]
Stunting				
Normal	337 [57%]	155 [51%]	182 [63%]	178 [57%]
Stunting	252 [43%]	146 [49%]	106 [37%]	137 [43%]
Wasting				
Normal	449 [77%]	230 [77%]	219 [77%]	236 [75%]
Wasting	135 [23%]	69 [23%]	66 [23%]	77 [25%]
Ever breastfed				
Yes	540 [91%]	271 [90%]	269 [92%]	279 [88%]
No	53 [9%]	30 [10%]	23 [8%]	38 [12%]
Early breastfeeding initiation				
Yes	518 [87%]	261 [86%]	257 [88%]	264 [83%]
No	77 [13%]	41 [14%]	36 [12%]	55 [17%]
MAIN EXPOSURE				
Mother social network				
Low	319 [54%]	168 [56%]	151 [52%]	—
High	274 [46%]	134 [44%]	140 [48%]	—
COVARIATES				
Child age				
0–6 months	71 [12%]	37 [12%]	34 [12%]	38 [12%]
7–24 months	238 [40%]	121 [40%]	117 [40%]	125 [39%]
Older than 24 months	286 [48%]	144 [48%]	142 [48%]	156 [49%]
Birth order				
First born	272 [46%]	125 [46%]	147 [54%]	151 [47%]
Second born	217 [37%]	115 [53%]	102 [47%]	103 [32%]
Third born or later	105 [18%]	61 [58%]	44 [42%]	65 [20%]
Maternal age				
13–19 years	9 [2%]	3[1%]	6 [2%]	3 [1%]
20–34 years	563 [95%]	288 [95%]	275 [94%]	306 [96%]
35 + years	22 [4%]	11 [4%]	11 [4%]	10 [3%]
Maternal occupation				
Housewife	536 [90%]	272 [90%]	264[90%]	279 [87%]
Other occupation	58 [10%]	30 [10%]	28 [10%]	40 [13%]
Maternal education				
No or lower education	98 [16%]	48 [16%]	50 [17%]	56 [18%]
Senior secondary school	292 [49%]	152 [50%]	140 [48%]	160 [50%]
Higher education	204 [34%]	102 [34%]	102 [35%]	103 [32%]

A strong maternal network significantly reduced the odds of the child never being breastfed (OR = 0.46, 95% CI = 0.24–0.85) and of delayed initiation of breastfeeding (OR = 0.42, 95% CI = 0.24–0.71). A strong maternal social network appeared to increase the risk of developing symptoms of anaemia compared to children whose mothers have a low social network level (OR = 1.82, 95% CI 1.31–2.56). After adjusting for covariates, these findings remained statistically significant. No significant associations with strong maternal networks were observed for stunting, wasting, underweight, low birthweight, or inappropriate introduction to solids. Table 2 reports the association between maternal social network and each nutritional outcome, presented as OR and 95% CI, including both the crude and the adjusted models, statistically significant findings highlighted in bold. Figure 2 visualizes these findings for illustrative purposes.

**Figure 2.**
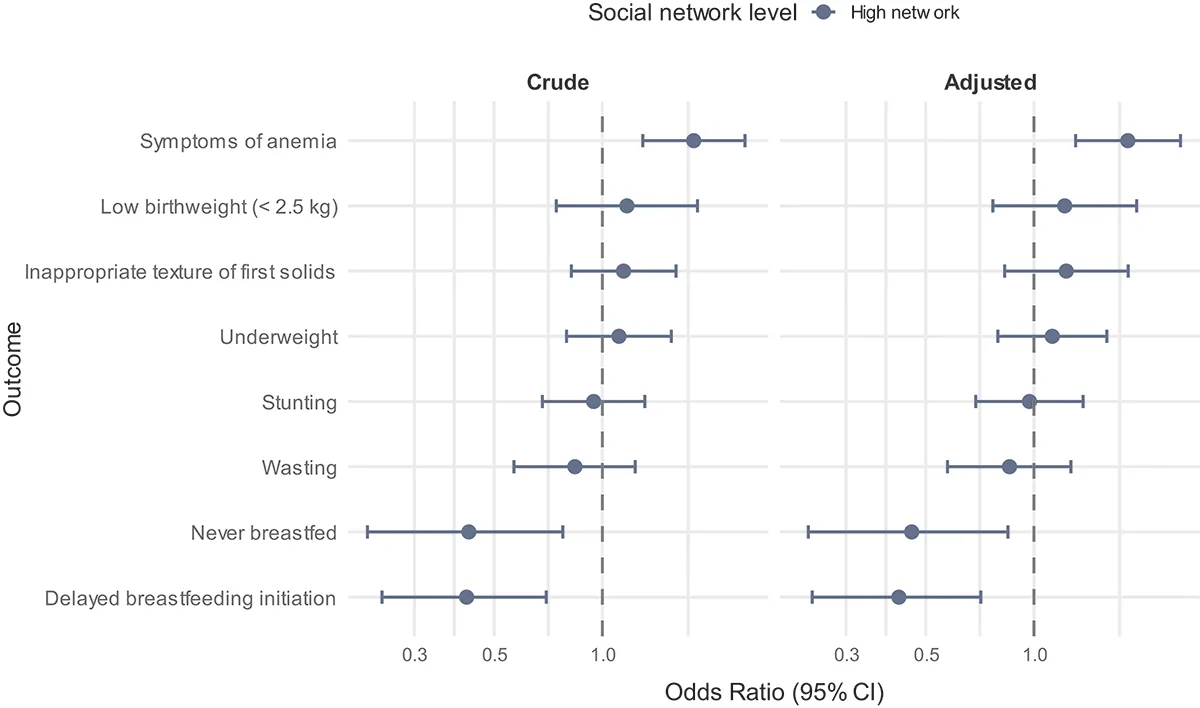
Association between maternal social network and child health related outcomes using data from HDSS, Belagavi, India 2024. Results of crude and adjusted logistic regression models for each outcome separately. The dots present OR and the horizontal lines crossing the dots illustrate the 95% CI.

**Table 2.** Association between maternal social network and child health-related outcomes using data from HDSS, Belagavi, India, 2024. Results of crude and adjusted logistic regression models for each outcome separately.

	Crude model OR [95%CI]	Adjusted model OR [95%CI]
CHILD HEALTH RELATED OUTCOMES		
Symptoms of anaemia		
No	1	1
Yes	1.79 [1.3–2.49]	**1.82 [1.31–2.56]**
Birthweight		
Normal [≥2.5 kg]	1	1
Low [<2.5 kg]	1.17 [0.74–1.84]	1.22 [0.77–1.93]
Introduction of first solids		
Appropriate	1	1
Inappropriate	1.15 [0.82–1.6]	1.23 [0.83–1.83]
Underweight		
Normal	1	1
Underweight	1.11 [0.79–1.56]	1.12 [0.79–1.59]
Stunting		
Normal	1	1
Stunting	0.95 [0.68–1.31]	0.97 [0.69–1.37]
Wasting		
Normal	1	1
Wasting	0.84 [0.57–1.23]	0.86 [0.57–1.27]
Ever breastfed		
Yes	1	1
No	0.43 [0.22–0.78]	**0.46 [0.24–0.85]**
Early breastfeeding initiation		
Yes	1	1
No	0.42 [0.24–0.7]	**0.42 [0.24–0.71]**

## Discussion

The main finding was the range of associations across all outcomes, indicating how they may be associated with the maternal social network and which outcomes were associated with the maternal social network in potentially favourable or unfavourable ways. A strong maternal social network was associated with lower odds of wasting, never breastfeeding, and delayed initiation of breastfeeding. However, it was associated with higher odds of being underweight, anaemia, inappropriate introduction of complementary foods, and low birthweight. For stunting, the association with maternal social networks was close to null. These associations between each nutritional outcome and the maternal social network, varying in both strength and direction, highlight the complexity of the effects of social networks on health.

The findings align with previous research on maternal social network’s and children’s health regarding undernutrition and breastfeeding where the maternal social network was protective [21,26]. Mothers may be influenced by their own network when deciding whether to breastfeed or to continue breastfeeding. It is then likely that a larger social network, as in the current study, may provide greater opportunities for social norms or include someone within the network that can provide the knowledge and support that the mother needs to breastfeed their child [26]. Similarly, early childhood feeding practices may also reflect the knowledge and practices of the maternal social network to some extent. Several influential actors in the maternal social network have previously been recognized, such as mothers, mother-in-laws, and friends, as well as health professionals and senior members of the community [27]. However, not all studies have reported consistent benefits of maternal social relationships for child nutritional outcomes. Previous research from LMICs has shown that improvements in maternal knowledge and social support do not always translate into improved child growth, suggesting that broader contextual and structural factors may modify these relationships [28,29]. This is consistent with the mixed pattern of associations observed in the present study. However, some findings suggested that a stronger maternal social network was associated with the inappropriate introduction of complementary foods, potentially leading to nutritional deficiencies. The result may reflect the poor knowledge and practices regarding complementary food among the close friends, or potentially social norms, within the social network of the mothers in Belagavi.

The only study from India on maternal social networks and child nutrition, conducted in Andhra Pradesh, reported lower odds of stunting among children whose mothers had larger social networks. Similarly, to this study, the definition of maternal social networks in the Indian study was based on the network size [21]. Our findings could neither support those results nor contradict them, as the odds ratio for stunting was close to 1, suggesting the association to be due to chance. The CI for all undernutrition categories (stunting, wasting, and underweight) were wide and included 1, and although not statistically significant, the point estimates consistently suggested lower odds of wasting and higher odds of underweight. These findings should be interpreted cautiously and considered hypothesis-generating rather than confirmatory. Wasting and underweight are both indicators of acute undernutrition conditions but maternal social networks seem to affect them in opposite directions. The difference between wasting and underweight is that while wasting is caused by recent weight loss due to infectious diseases or simply shortage of food, being underweight can also have a chronic component, and stunted children may be underweight [12]. One possible explanation for the different direction of association for waste and underweight could be that larger networks may provide information and access to resources but can also dilute resources. Information about hygiene and prevention of infectious diseases, or access to food through a larger network may be associated with lower odds of wasting. Diluting resources within a larger network could potentially result in larger maternal social networks being less protective against chronic categories of malnutrition. All these assumptions depend on the structure of the social network and what services the social network can provide through its different pathways. While this study measured maternal social network size, the functional characteristics of these networks remain unknown.

The current study also found evidence of a strong relationship between maternal networks and childhood anaemia, an association which has not been found in the previous literature, and little was found supporting these results. Risk factors for childhood anaemia have previously been investigated in India but did not include maternal social networks as an independent variable [30,31]. Neither maternal age nor education affected the association; however, household wealth and maternal anaemia were not investigated due to logistical reasons. Maternal anaemia can result in low iron levels in breastmilk, and breastfeeding children may be at risk for iron deficiency and childhood anaemia [31]. It has also been suggested that anaemia, as well as undernutrition, is more likely when scarce resources need to be shared, such as in larger and poorer households [32]. However, household size can in some settings also be protective, and living with paternal grandparents can possibly decrease the risk of anaemia [33]. This suggests that depending on how the maternal social network is defined, the results can differ when it comes to having anaemia as an outcome.

The study’s main strength is its easily replicable design, which enables clear comparisons between each child’s health outcome and maternal social network. This enabled a consistent comparison of the association between maternal social network size, and multiple child health outcomes. Also, data was collected in several villages in rural villages, providing a comprehensive picture of this marginalized rural population.

Some limitations should also be taken into account. First, household level factors such as the sex of the household head or household size could not be considered as covariates as the dataset did not include these variables. Second, this study had a relatively small sample size, which may have affected the estimates and the significance of the results. The small sample size also affected the division of maternal social network and may have masked some information when dichotomizing the variables this way. Our measure may not fully capture the multidimensional nature of maternal social networks, but the results nevertheless provide useful hypotheses for future research. Third, one of the main findings mentioned was the strong odds of anaemia for children with a strong maternal network. The classification of anaemia was based on data collectors’ examination of the children, and not based on a clinical definition. Many mothers objected to having their children’s blood sampled, making haemoglobin measurements infeasible. Consequently, anaemia was assessed using observable clinical signs rather than laboratory confirmation. Although pallor and koilonychia are commonly used clinical indicators, they are less accurate than haemoglobin measurements and may have resulted in non-differential misclassification of the outcome. Therefore, the findings related to anaemia should be interpreted with caution. Also, recall bias must also be considered as a limitation for this study as the data is mostly based on information from the mothers, who may have remembered some information incorrectly, affecting the reliability of the data. The limitations of this study may have contributed to the few statistically significant findings which call for additional research on this topic. An improved study would consider additional confounding factors, particularly household wealth, maternal nutritional status (including maternal anaemia), and household size. These factors are well-established determinants of child nutrition and may also be associated with maternal social networks. Consequently, residual confounding cannot be excluded, and some observed associations may have been overestimated, underestimated, or partly explained by these unmeasured factors. The findings should therefore be interpreted as associations rather than evidence of independent effects of maternal social networks [15]. Finally, the cross-sectional design precludes establishing temporal relationships or causal inferences between maternal social networks and child health outcomes. Although maternal social networks are likely to influence caregiving practices and child nutrition, reverse causation cannot be excluded.

These findings highlight the need for further studies, ideally with larger sample sizes, more comprehensive measures of maternal social networks, improved assessment of outcomes (particularly anaemia), and qualitative methods to better understand the observed associations. Future interventions should consider the potential differential effects that strengthening maternal social networks may have on child nutrition. This will enable these interventions to address child nutrition more effectively and limit the possibly negative effects on children’s health.

## Conclusion

This study hypothesized that a strong maternal social network would benefit children’s health and nutrition. Some contradictory findings, such as the higher odds of anaemia among children whose mothers had strong social networks, were unexpected. In conclusion, maternal social networks were associated with both favourable and unfavourable child nutritional outcomes. Strengthening maternal social connectedness may improve some outcomes, such as breastfeeding practices, but may not benefit all aspects of child nutrition. Future interventions should therefore consider the complexity of maternal social networks and their potentially differential associations with child health outcomes.

## Data Availability

The data are not publicly available, but access for replication analyses is possible upon legitimate request. Requests to access the datasets should be directed to the corresponding author.
